# Risk Factors for Death from Invasive Pneumococcal Disease, Europe, 2010

**DOI:** 10.3201/eid2103.140634

**Published:** 2015-03

**Authors:** Adoración Navarro-Torné, Joana Gomes Dias, Frantiska Hruba, Pier Luigi Lopalco, Lucia Pastore-Celentano, Andrew J. Amato Gauci, Invasive Pneumococcal Disease Study Group

**Affiliations:** Universidad Autónoma de Barcelona, Barcelona, Spain (A. Navarro-Torné);; European Centre for Disease Prevention and Control, Stockholm, Sweden (A. Navarro-Torné, J. Gomes Dias, F. Hruba, P.L. Lopalco, L. Pastore-Celentano, A.J. Amato Gauci)

**Keywords:** Streptococcus pneumonia, invasive pneumococcal disease, death, pneumococcal vaccine, meningitis, antimicrobial drug resistance, Europe, bacteria

## Abstract

We studied the possible association between patient age and sex, clinical presentation, *Streptococcus pneumoniae* serotype, antimicrobial resistance, and death in invasive pneumococcal disease cases reported by 17 European countries during 2010. The study sample comprised 2,921 patients, of whom 56.8% were men and 38.2% were >65 years of age. Meningitis occurred in 18.5% of cases. Death was reported in 264 (9.0%) cases. Older age, meningitis, and nonsusceptibility to penicillin were significantly associated with death. Non–pneumococcal conjugate vaccine (PCV) serotypes among children <5 years of age and 7-valent PCV serotypes among persons 5–64 years of age were associated with increased risk for death; among adults >65 years of age, risk did not differ by serotype. These findings highlight differences in case-fatality rates between serotypes and age; thus, continued epidemiologic surveillance across all ages is crucial to monitor the long-term effects of PCVs.

## Abstract 2

Risk varies by *Streptococcus pneumoniae* serotype.

*Streptococcus pneumoniae* causes severe invasive disease that results in considerable illness and death. The incidence of invasive pneumococcal disease (IPD) is higher during the early years of life and among elderly persons (*1*). Geographic and ethnic differences also exist (*1*,*2*). Environmental factors (i.e., ambient temperature, humidity, and air pollution) affect IPD incidence (*3*,*4*). IPD has also been related to recent respiratory viral infection (*4*).

The ability of the different *S. pneumoniae* serotypes to cause disease has been related to serotype-specific characteristics and the molecular size of the capsular polysaccharide and chemical composition, among other factors (*5*). Therefore, it seems plausible that different serotypes exhibit different virulence and propensity to cause certain clinical presentation (*5*).

Brueggemann et al. studied the invasive disease potential of different *S. pneumoniae* serotypes (*6*). They concluded that so-called “highly invasive” serotypes (including 4, 1, 14, 18C, and 7F), convey a higher risk for invasive disease than do the “low invasive” serotypes (including 3, 15B/C, and 6B), which are more frequently isolated as colonizers (*7*). Furthermore, serotype distribution varies with patient age, both in disease and in nasopharyngeal colonization (*2*,*8*–*10*). However, evidence exists that pneumococcal invasiveness does not necessarily mean lethality (*7*). Low invasive serotypes usually account for higher case-fatality rates (CFRs).

The availability of 7-valent, 10-valent, and 13-valent pneumococcal conjugate vaccines (PCV7, PCV10, and PCV13, respectively) and their introduction as part of national immunization schedules have contributed to reducing illnesses and death from IPD (*10*–*12*). Nevertheless, the subsequent replacement of vaccine serotypes by nonvaccine serotypes is an accepted and global phenomenon (*13*,*14*).

The incidence of drug- and multidrug-resistant *S. pneumoniae* strains is increasing worldwide (*15*). Antimicrobial use and abuse is a main driver for the emergence of antimicrobial resistance in respiratory pathogens. Persons who carry (nasopharyngeal colonization), and hence share the potential to transmit resistant pneumococci, also are more susceptible to IPD caused by resistant strains (*16*).

Monitoring antimicrobial resistance trends and serotype distribution is paramount because this information is essential in helping to determine risk factors and optimizing the appropriate clinical management of cases and public health interventions. We studied the possible association between age, sex, serotype, clinical presentation, antimicrobial resistance, and death among persons reported to have IPD in European countries during 2010.

## Materials and Methods

### Data

IPD data derived from passive national surveillance case notification systems were collected during 2010 by 26 European Union (EU)/European Economic Area countries (Austria, Belgium, Bulgaria, Cyprus, Czech Republic, Denmark, Estonia, Finland, France, Greece, Hungary, Iceland, Ireland, Italy, Latvia, Lithuania, Malta, Netherlands, Norway, Poland, Romania, Slovakia, Slovenia, Spain, Sweden, and United Kingdom); data were submitted to The European Surveillance System. The platform of The European Surveillance System is a metadata-driven system for the collection, validation, cleaning, and analysis of data hosted by the European Centre for Disease Prevention and Control. Surveillance systems differ across Europe, and data were reported with varying levels of completeness. Countries reported only laboratory-confirmed cases based on the EU 2008 case definition.

### Study Sample

**Figure 1:**
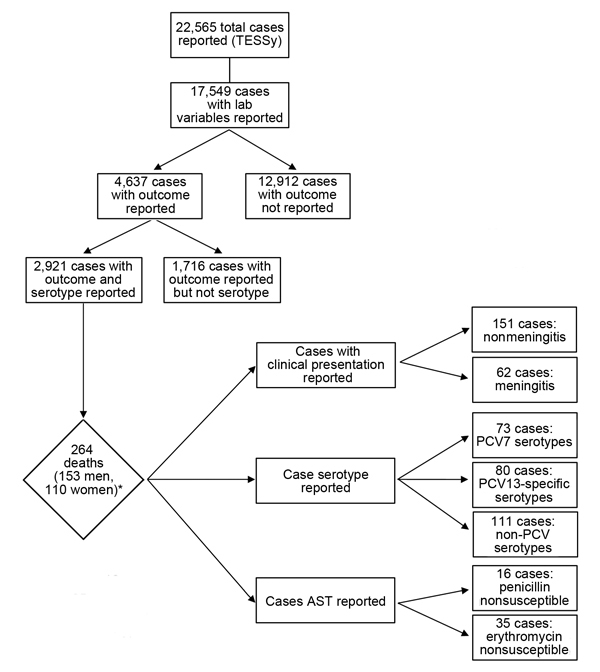
Flow of invasive pneumococcal disease cases through the study, Europe, 2010. *Sex was unknown for 1 patient. AST, antimicrobial susceptibility testing; PCV, pneumococcal conjugate vaccine; PCV7, 7-valent PCV; PCV13, 13-valent PCV; TESSy, The European Surveillance System.

The study sample was the subsample of cases for which information was available about both serotype and outcome (Figure 1). The sample represents data from 17 European countries (Table 1).

**Table 1:** Characteristics of patients with invasive pneumococcal disease, Europe, 2010*

Characteristic	No. cases (% of total), N = 17,549	Sample size, no. (%), n = 2,921†
Sex		
F	7,915 (45.3)	1,257 (43.2)
M	9,565 (54.7)	1,651 (56.8)
Age group, y		
<5	1,980 (11.3)	570 (19.7)
5–64	7,819 (44.7)	1,222 (42.1)
>65	7,684 (44.0)	1,108 (38.2)
Outcome		
Nonfatal	4,146 (89.4)	2,657 (91.0)
Fatal	491 (10.6)	264 (9.0)
Clinical presentation		
Nonmeningitis	6,047 (79.4)	1,722 (81.5)
Meningitis	1,572 (20.6)	391 (18.5)
Serotype		
PCV13-specific‡	4,185 (42.1)	1,235 (42.7)
PCV7	1,772 (17.8)	517 (17.9)
Non-PCV	3,989 (40.1)	1,137 (39.4)
Antimicrobial susceptibility		
Penicillin		
Susceptible	8,420 (91.1)	1,949 (94.1)
Nonsusceptible§	827 (8.9)	122 (5.9)
Erythromycin		
Susceptible	6,911 (82.5)	1,573 (76.4)
Nonsusceptible	1,471 (17.5)	486 (23.6)

*Numbers do not add to the total in each category because of missing data. See Figure 1. PCV, pneumococcal conjugate vaccine; PCV7, 7-valent PCV; PCV13, 13-valent PCV. †Defined as patients for whom information was available about serotype and outcome. ‡Serotypes contained in PCV13 but not in PCV7. §Either resistant or intermediate resistance.

### Study Variables

An episode of IPD was defined as the isolation of a strain or detection of nucleic acid or antigen of *S. pneumoniae* from a normally sterile site. Countries reported IPD outcome according to their national surveillance and guidelines. The following age groups were defined for the study: <5 years, 5–64 years, and >65 years. For purpose of this analysis, clinical presentation was recoded as “meningitis” and “nonmeningitis.” Clinical presentation was grouped on the basis of a literature review (*5*), which suggested that meningitis and nonmeningitis had different degrees of severity and conveyed different rates of death.

Serotypes were grouped into 3 categories: PCV7 serotypes (serotypes in PCV7: 4, 6B, 9V, 14, 18C, 19F, and 23F), PCV13-specific serotypes (serotypes in PCV13 but not in PCV7: 1, 3, 5, 6A, 7F, and 19A), and non-PCV serotypes (serotypes not in any PCV). Results of antimicrobial susceptibility testing to penicillin and erythromycin were reported as “susceptible,” “intermediate,” or “resistant” by the countries according to their national standards and protocols. Therefore, information was not available about the breakpoints and guidelines used for antimicrobial susceptibility testing in each country. For example, in the European Antimicrobial Resistance Surveillance Network report for 2010 (*17*), 66% of reporting laboratories in Europe used Clinical and Laboratory Standards Institute standards, whereas 29% applied the European Committee on Antimicrobial Susceptibility Testing guidelines.

For this study, we redefined the variable to include just 2 categories: “susceptible” (cases reported as susceptible by the countries) and “nonsusceptible” (intermediate and resistant), both for penicillin and erythromycin. Methods for the characterization of isolates and for antimicrobial susceptibility testing are provided in detail in the 2010 IPD enhanced surveillance report by the European Centre for Disease Prevention and Control (*18*).

### Statistical Analysis

Categorical variables are presented as number of cases and percentages. We used the Pearson χ^2^ test to compare the proportion of deaths by PCV7, PCV13-specific, and non-PCV serotypes; the proportion of deaths by the defined age groups and by sex; the proportion of deaths by clinical presentation; and the proportion of deaths in antimicrobial-susceptible and -nonsusceptible cases, according to antimicrobial drug type. We used the Fisher exact test to analyze the association between penicillin-susceptible/penicillin-nonsusceptible IDP and outcome for patients <5 years of age and non-PCV serotypes and to assess differences between penicillin-susceptible/penicillin-nonsusceptible cases and outcome for serotype 35B. In addition, we assessed the associations between each serotype and death using a generalized linear model with log-link function. This analysis was performed for the 28 serotypes that accounted for up to 80% of cases with fatal outcomes; each individual serotype was also compared with all the others.

Univariable analysis was performed for the 264 fatal cases to identify factors associated with a fatal outcome. To test the association between age, serotype, clinical presentation, and death, a generalized linear model with robust SEs accounting for the country effect was fitted because data came from different national surveillance systems and vaccination policies and practices differ widely across Europe. We studied the role of variables as potential confounders/modifiers, but only age was statistically significant. Age was an effect modifier of the association between serotype and risk for death, and thus the analysis was stratified by age group.

We also conducted regression analysis. The regression model comprised factors that were significant by univariable analysis and that had previously been hypothesized to affect IPD CFRs.

All p values were 2 tailed, and statistical significance was defined as p<0.05. We conducted statistical analyses by using STATA 12.0 (StataCorp, College Station, TX, USA).

## Results

### Case Characteristics

In 2010, the European countries reported 22,565 IPD cases. Of these, information was available about laboratory variables for 17,549 cases (Figure 1); outcome was known for 4,637 of these. The study sample comprised 2,921 cases for which information was available about serotype and outcome.

A total of 56.8% of cases (Table 1) occurred in men, and 38.2% of cases were among adults >65 years of age. Children <5 years of age accounted for 19.7% of cases. A total of 264 (9.0%) persons died. Meningitis occurred in 18.5% of cases. PCV13-specific serotypes (1, 3, 5, 6A, 7F, 19A) accounted for 42.7% of cases. Nonsusceptibility (intermediate + resistant) to penicillin was reported in 122 (5.9%) of 2,071 cases; nonsusceptibility to erythromycin was reported in 486 (23.6%) of 2,059 cases (Table 1).

**Figure 2:**
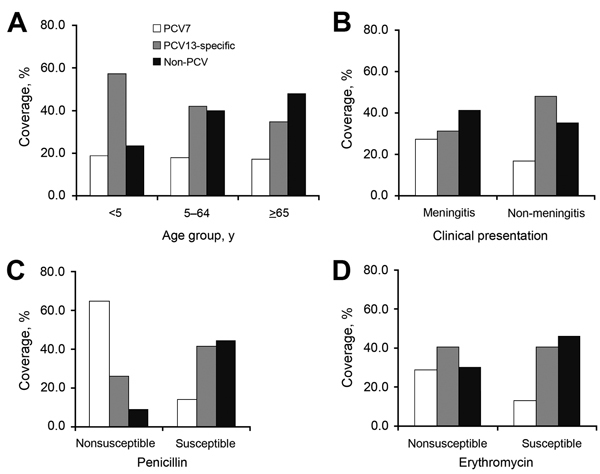
Invasive pneumococcal disease study variables and PCV coverage of *Streptococcus pneumoniae* serotypes, Europe, 2010. A) Age group. B) Clinical presentation. C) Penicillin susceptible. D) Erythromycin susceptible. For all 4 variables, p<0.001. White bars, PCV7 serotypes; gray bars, PCV13 serotypes; black bars, non-PCV serotypes. PCV, pneumococcal conjugate vaccine; PCV7, 7-valent PCV; PCV13, 13-valent PCV.

PCV13-specific serotypes caused 57.7% (p<0.001) of cases among children <5 years of age (Figure 2). Non-PCV serotypes accounted for 48.0% of cases among adults >65 years of age. Meningitis cases were predominantly caused by non-PCV serotypes (41.4%, p<0.001) (Figure 2). Nonsusceptibility to penicillin was highest among PCV7 serotypes (64.8%, p<0.001) (Figure 2).

The Pearson χ^2^ analysis (Table 2) demonstrated a lack of statistical association between sex and death (p = 0.631). The CFR was highest for adults >65 years of age (13.7%, p<0.001); 2.3% of children <5 years of age died.

**Table 2:** Associations between invasive pneumococcal disease study variables and death, Europe, 2010*

Variable	Outcome		p value†
	Nonfatal, no. (%)	Fatal, no. (%)	
Sex			
F	1,147 (91.3)	110 (8.8)	0.631
M	1,498 (90.7)	153 (9.3)	
Age group, y			
<5	557 (97.7)	13 (2.3)	
5–64	1,123 (91.9)	99 (8.1)	<0.001
>65	956 (86.3)	152 (13.7)	
Clinical presentation			
Nonmeningitis	1,571 (91.2)	151 (8.8)	<0.001
Meningitis	329 (84.1)	62 (15.9)	
Serotype			
PCV13-specific‡	1,155 (93.5)	80 (6.5)	<0.001
PCV7	444 (85.9)	73 (14.1)	
Non-PCV	1,028 (90.5)	111 (9.5)	
Antimicrobial susceptibility			
Penicillin			
Susceptible	1,815 (93.1)	134 (6.9)	
Nonsusceptible§	106 (86.9)	16 (13.1)	
Erythromycin			0.010
Susceptible	1,464 (93.1)	109 (6.9)	
Nonsusceptible	451 (92.8)	35 (7.2)	

*PCV, pneumococcal conjugate vaccine; PCV7, 7-valent PCV; PCV13, 13-valent PCV; †Pearson χ^2^ test. ‡Serotypes contained in PCV13 but not in PCV7. §Either resistant or intermediate resistance.

Clinical presentation was associated with death. The CFR for persons with meningitis was 15.9% compared with 8.8% for those without meningitis (p<0.001).

Death was also associated with nonsusceptibility to penicillin. Death occurred in 13.1% of cases in which *S. pneumoniae* was not susceptible to penicillin (p = 0.010) (Table 2). Nonsusceptibility to erythromycin was not significantly associated with death (p = 0.837).

We determined the association between individual serotype and death (Table 3). Serotype 35B (relative risk [RR] 4.98, 95% CI 2.49–9.95), serotype 4 (RR 2.03, 95% CI 1.04–3.95), and serotype 11A (RR 1.97, 95% CI 1.33–2.94) were most associated with death. Serotype 3 (RR 1.39, 95% CI 0.88–2.21) accounted for the highest number and the highest percentage (13.3%) of serotype-specific deaths, but the association with death was not statistically significant (p = 0.161). In contrast, for serotype 1 (RR 0.25, 95% CI 0.13–0.48) and serotype 5 (RR 0.15, 95% CI 0.09–0.26), the association with death was significant. Subanalysis of the association between susceptibility to penicillin and outcome for serotype 35B found no significant differences in risk for death between susceptible and nonsusceptible cases.

**Table 3:** *Streptococcus pneumoniae* serotype in invasive pneumococcal disease and association with death, Europe, 2010*

Serotype	PCV†	Fatal, %	Nonfatal, %	RR (95% CI)	p value‡
3	PCV13-specific§	13.3	9.6	1.39 (0.88–2.21)	0.161
**4**	**PCV7**	**6.1**	**2.8**	**2.03 (1.04–3.95)**	**0.038**
19A	PCV13-specific	6.1	7.6	0.80 (0.41–1.57)	0.515
14	PCV7	5.7	4.6	1.23 (0.78–1.85)	0.369
7F	PCV13-specific	4.9	8.3	0.59 (0.35–1.01)	0.053
6B	PCV7	3.8	1.7	2.01 (0.79–5.16)	0.144
19F	PCV7	3.8	1.9	1.85 (0.93–3.65)	0.078
22F	Non-PCV	3.8	2.8	1.35 (0.89–2.03)	0.157
9V	PCV7	3.4	2.2	1.50 (0.95–2.38)	0.081
23F	PCV7	3.4	2.3	1.42 (0.60–3.32)	0.423
**1**	**PCV13-specific**	**3.4**	**13.1**	**0.25 (0.13–0.48)**	**<0.001**
**11A**	**Non-PCV**	**2.3**	**1.1**	**1.97 (1.33–2.94)**	**0.001**
10A	Non-PCV	2.3	1.4	1.52 (0.86–2.68)	0.147
6A	PCV13-specific	2.3	2.3	1.01 (0.39–2.57)	0.990
6C	Non-PCV	1.9	0.7	2.33 (0.93–5.86)	0.072
9N	Non-PCV	1.9	1.5	1.21 (0.52–2.82)	0.664
12F	Non-PCV	1.9	1.8	1.07 (0.51–2.23)	0.867
**35B**	**Non-PCV**	**1.5**	**0.2**	**4.98 (2.49–9.95)**	**<0.001**
33F	Non-PCV	1.5	0.9	1.53 (0.55–4.28)	0.414
18C	PCV7	1.5	1.2	1.23 (0.40–3.76)	0.713
8	Non-PCV	1.5	3.1	0.59 (0.25–1.06)	0.073
23A	Non-PCV	1.1	0.7	1.51 (0.66–3.45)	0.323
15A	Non-PCV	0.8	0.7	1.05 (0.46–2.43)	0.909
15B	Non-PCV	0.8	1.0	0.79 (0.26–2.41)	0.677
24F	Non-PCV	0.4	0.6	0.69 (0.12–4.09)	0.683
**5**	**PCV13-specific**	**0.4**	**2.6**	**0.15 (0.09–0.26)**	**<0.001**

*PCV, pneumococcal conjugate vaccine; PCV7, 7-valent PCV; PCV13, 13-valent PCV; RR, relative risk. Boldface indicates statistical significance. †Classification of serotypes according to study group. ‡Generalized linear model with log-link function. §Serotypes contained in PCV13 but not in PCV7.

### Risk Factors for IPD-Associated Death

Univariable analysis showed differences between nonfatal and fatal cases (Table 4). Persons 5–64 years of age (RR 3.55, 95% CI 1.66–7.61) and >65 years of age (RR 4.79, 95% CI 3.08–11.76) had a higher risk for death than did children <5 years of age. In the univariable analysis, meningitis (RR 1.81, 95% CI 1.25–2.61, p = 0.002) was significantly associated with death. PCV7 serotypes were also significantly associated with death (RR 2.18, 95% CI 1.06–4.48, p = 0.034). Conversely, non-PCV serotypes were not associated with death (RR 1.47, 95% CI 0.94–2.28).

**Table 4:** Association between invasive pneumococcal disease study variables and death, Europe, 2010*

Variable	Relative risk† (95% CI)
Sex	
F	Reference
M	1.06 (0.88–1.28)
Age group, y	
<5	Reference
5–64	3.55 (1.66–7.61)
>65	4.79 (3.08–11.76)
Clinical presentation	
Nonmeningitis	Reference
Meningitis	1.81 (1.25–2.61)
Serotype	
PCV13-specific‡	Reference
PCV7	2.18 (1.06–4.48)
Non-PCV	1.47 (0.94–2.28)
Antimicrobial susceptibility	
Penicillin	
Susceptible	Reference
Nonsusceptible§	1.91 (1.16–3.13)
Erythromycin	
Susceptible	Reference
Nonsusceptible	1.04 (0.84–1.29)

*PCV, pneumococcal conjugate vaccine; PCV7, 7-valent PCV; PCV13, 13-valent PCV.. †Generalized linear model with log-link function. ‡Serotypes contained in PCV13 but not in PCV7. §Either resistant or intermediate resistance.

Nonsusceptibility to penicillin was associated with increased risk for death (RR 1.91, 95% CI 1.16–3.13). Nonsusceptibility to erythromycin was not significantly associated with death (RR 1.04, 95% CI 0.84–1.29).

Our comparison of susceptibility to penicillin and outcome for clinical presentation showed that the association with the outcome remained statistically significant only for meningitis cases (RR 1.82, 95% CI 1.27–2.62, p = 0.001). These factors were not associated with nonmeningitis cases (RR 1.31, 95% CI 0.28–6.01).

Age was an effect modifier. In the stratified analysis, we found that among children <5 years of age, risk for death from non-PCV serotypes increased (RR 3.68, 95% CI 1.27–10.69) (Table 5), whereas among persons 5–64 years of age, PCV7 serotypes conveyed the highest risk for death (RR 2.68, 95% CI 1.37–5.23). Among adults >65 years of age, risk for death among the serotypes did not differ significantly.

**Table 5:** Stratified analysis of *Streptococcus pneumoniae* serotype distribution in a study of invasive pneumococcal disease, Europe, 2010*

Age group, y	Survived, no. (%)	Died (%)	RR (95% CI)	p value
<5				
PCV13-specific	325 (98.8)	4 (1.2)	1	
PCV7	104 (97.2)	3 (2.8)	2.31 (0.35–15.02)	0.382
Non-PCV	128 (95.5)	6 (4.5)	3.68 (1.27–10.69)	0.017
5–64				
PCV13-specific	486 (94.4)	29 (5.6)	1	
PCV7	186 (84.9)	33 (15.1)	2.68 (1.37–5.23)	0.004
Non-PCV	451 (92.4)	37 (7.6)	1.35 (0.64–2.82)	0.429
>65				
PCV13-specific	338 (87.8)	47 (12.2)	1	
PCV7	154 (80.6)	37 (19.4)	1.59 (0.90–2.79)	0.108
Non-PCV	464 (87.2)	68 (12.8)	1.05 (0.64–1.72)	0.856

*PCV, pneumococcal conjugate vaccine; PCV7, 7-valent PCV; PCV13, 13-valent PCV; RR, relative risk

We analyzed the association between susceptibility to penicillin and outcome for non-PCV serotypes. Children <5 years of age showed no differences between susceptible and nonsusceptible cases.

## Discussion

Our analysis of IPD surveillance data from Europe in 2010 unveiled a significant association between death and older age, meningitis, serotypes contained in PCV7, and nonsusceptibility to penicillin. As have many other studies, we found an association between increased age and death (*19*–*22*). The risk was higher for adults >65 years of age (RR 4.79, 95% CI 3.08–11.76) than for persons 5–64 years of age (RR 3.55, 95% CI 1.66–7.61). However, the lack of information about patients’ clinical characteristics impedes accurate assessments of these differences.

Elderly persons have been postulated to have an increased susceptibility to—in addition to co-ocurring conditions—pneumococcal disease because of reduced splenic function (*23*), age-related changes in respiratory tract, immunosenescence, and cellular senescence related to age-associated inflammation (*23*). The higher incidence and death rates for IPD in this age group is remarkable and highlights the need to direct vaccination toward the elderly. These findings may present an opportune moment to revisit adult vaccination recommendations and programs in European countries (*24*).

We did not find sex to be significantly associated with death. However, other studies have shown association either with men (*25*) or women (*23*,*26*).

In our study, presence of meningitis was significantly associated with death. Harboe et al. obtained similar results in a large population-based cohort study (*25*). In Denmark, another study concluded that patients with pneumococcal meningitis had increased death rates, but these rates derived from severe underlying conditions (*27*). CFRs for pneumococcal meningitis are usually higher than for nonmeningitis (*28*). More recently, Ladhani et al. found that the CFR was higher for children with meningitis in England and Wales (*29*). This study showed that infecting serotype was not associated with death (*29*), whereas meningitis and co-occurring conditions were significantly associated with death. In our analysis, meningitis was predominantly caused by non-PCV serotypes; this finding could be an effect of PCV introduction, as observed in other studies (*30*). Another analysis of susceptibility to penicillin by clinical presentation showed a higher risk for death among persons with nonsusceptible IPD than for those with susceptible IPD who had meningitis. Therefore, in the absence of information about clinical management of cases and existing co-occurring conditions, the association between meningitis and nonsusceptibility to penicillin might be an explanation.

Capsular differences between serotypes affect clinical presentation and outcome (*10*,*31*,*32*). These differences are in accordance with our study, which found PCV7 serotypes were associated with death in the univariable analysis. Among children <5 years of age, PCV13-specific serotypes were most frequently identified, compared with PCV7 and non-PCV serotypes, as defined in our study. In 2010, PCV13 was already licensed, and many European countries began moving from PCV7 to the higher-valent vaccine, although with different schemes, policies, and dates of introduction. Nevertheless, these changes are unlikely to have affected our study findings because we analyzed data from 2010.

After stratification, the highest risk for death among children <5 years of age corresponded to non-PCV serotypes. This finding could be attributed to serotype replacement after pneumococcal vaccination (*29*,*30*). Our analysis found no differences between penicillin-susceptible and -nonsusceptible cases among children <5 years of age and non-PCV serotypes subgroup with respect to death. However, the overall percentage of meningitis cases was high (18.5% of the study sample), and meningitis was predominantly caused by non-PCV serotypes (p<0.001) (Figure 2). Hence, vaccines with enhanced serotype coverage (higher valency) might be needed to prevent IPD in this age group in the near future. 

Among persons 5–64 years of age, the risk for death was highest for PCV7 serotypes, which were predominantly nonsusceptible to penicillin (p<0.001) (Figure 2). Reductions in IPD caused by PCV7 serotypes in non–vaccine-eligible age groups in countries with universal use of PCV7 might indicate the indirect effect of PCV7 (*33*). However, because vaccine policies differed among European countries at the time of the study, this indirect effect might not be reflected in the pooled data (Table 6).

**Table 6:** Characteristics of national pneumococcal vaccination programs in European Union/European Economic Area countries, 2010*

Country	Date of PCV7 introduction	Scope of PCV vaccination program	Immunization schedule	Dose				Vaccine coverage†	Year of measurement
				First, mo	Second, mo	Third, mo	Fourth, mo		
Austria	2004 Jul	Universal	3+1 dose	3	5	7	12–24	NA	NA
Belgium	2005 Jan	Universal	2+1 dose	2	4	12		97	2010
Bulgaria	2010 Apr	Universal	3+1 dose/2+1 dose	2	3	4	12	NA	NA
Cyprus	2008 Aug	Universal	3+1 dose	2	4	6	12–15	NA	NA
Czech Republic	2010 Jan	Risk-based	3+1 dose	2	4	6	18	86.3	2010
Denmark	2007 Oct	Universal	2+1 dose	3	5	12		85	2010
Estonia	NA	NA	not decided	NA	NA	NA	NA	NA	NA
Finland	2009 Jan	Risk-based	2+1 dose	3	5	12		NA	NA
France	2006 Jun	Universal	2+1 dose	2	4	12		81	2008
Germany	2006 Jul	Universal	3+1 dose	2	3	4	11–14	52.9	2010
Greece	2006 Jan	Universal	3+1 dose	2	4	6	12–15	NA	NA
Hungary	2008 Oct	Universal	2+1 dose	2	4	15		81.1	2009
Iceland	2006 Dec	Risk-based	2+1 dose	3	5	12		NA	NA
Ireland	2002 Oct	Universal	2+1 dose	2	6	12		89	2009
Italy	2005 May	Universal/risk- based	2+1 dose	3	5	11		55	2008
Latvia	2010 Jan	Universal	3+1 dose	2	4	6	12–15	51	2010
Lithuania	NA	NA	3+1 dose	2	4	6	24	NA	NA
Luxembourg	2003 Feb	Universal	3+1 dose	2	3	4	12–15	86	2010
Malta	2007 Jan	Risk-based	3+1 dose	2	4	13	None	NA	NA
Netherlands	2006 Jun	Universal	3+1 dose	2	3	4	11	94	2009
Norway	2006 Jul	Universal	2+1 dose	3	5	12		90	2009
Poland	2008 May	Risk-based	3+1 dose/2+1 dose	NA	NA	NA	NA	1.70	2008
Portugal	2010 Jun	Risk-based	2+1 dose	2	4	12–15		52	2009
Romania‡			3+1 dose	2	4	6	15–18		
Slovakia§	2006 Jan	Risk-based	2+1 dose	2	4	10		99.2	2009
Slovenia	2005 Sep	Risk-based	3+1 dose	2-3	4	6	24	NA	NA
Spain¶	2001 Jun	Risk-based	3+1 dose	2	4	6	15	NA	NA
Sweden	2009 Jan	Universal	2+1 dose	3	5	12		NA	NA
United Kingdom	2006 Sep	Universal	2+1 dose	2	4	13		90	2010

*NA, not available; PCV, pneumococcal conjugate vaccine; PCV7, 7-valent PCV. Blank cells indicate not applicable. †Sources: Vaccine European New Integrated Collaboration Effort II project and World Health Organization estimates of PCV7 coverage. ‡PCV7 was registered in September 2007 for voluntary use on a private basis. §Universal as of April 2008. ¶Universal introduction in the autonomous region of Madrid in November 2006.

Serotypes 1, 5, and 7F have been described as having high potential for invasiveness (these serotypes are carried for a short time) but are associated with milder disease and lower CFRs (*7*,*9*,*19*,*34*). As in those studies, we found that serotypes 1 and 5 caused IPD but were not associated with death.

Serotype 35B has been reported as nonsusceptible to penicillin (*35*). The subanalysis on susceptibility to penicillin for serotype 35B showed that penicillin nonsusceptibility did not affect the risk for death for serotype 35B. Nevertheless, the increased risk for death of non-PCV serotypes 11A and 35B merits further monitoring.

We found penicillin nonsusceptibility to be significantly associated with death, as described by others (*20*,*36*). Nevertheless, in other large studies, this association was not found (*21*,*26*,*34*,*37*), and the effect of multidrug-resistant strains remains to be determined. Conversely, we found that erythromycin nonsusceptibility did not significantly affect death, as described by Song et al. (*37*) and Martens et al. (*20*). A plausible explanation might be the additional benefits of macrolides (i.e., their immunomodulatory/antiinflamatory properties), which might be important when these drugs are used in combination with other therapeutic agents (*38*).

Antimicrobial resistance to *S. pneumoniae* is increasing in many countries in Europe (*17*), and the prudent use of antibacterial drugs, apart from immunization, is pivotal in preventing and controlling IPD. Furthermore, these findings underpin the importance of antimicrobial susceptibility testing to assist with the clinical management of cases and to provide data on prevalence of antimicrobial resistance.

The major strength of our study is its large sample size; data were from national surveillance systems across Europe (i.e., we analyzed IPD individual-level data from populations in a large geographic area). In 2010, European IPD surveillance collected data corresponding to ≈82% of the total population of EU/European Economic Area countries. This enhanced surveillance for IPD data pooled together at supranational level enables comparisons with other parts of the world.

Despite its strengths, our study has some limitations. Surveillance of IPD varies markedly in Europe, including differences in laboratory methods to confirm cases, reporting, and medical practices. Therefore, a certain degree of underdiagnosis and underreporting are likely to exist in this dataset. Moreover, surveillance systems for IPD differ in sensitivity, representativeness, and specificity across Europe; these variations might have influenced the results because some countries were major contributors (Table 7) and ascertainment bias might have also occurred. Information about co-occurring conditions or clinical management of cases that might have affected outcome was also missing. European countries introduced pneumococcal vaccination at different times and with different policies, which might have affected the serotype distribution throughout Europe. Furthermore, the incomplete information about the vaccination status of cases makes difficult the interpretation of results. These limitations emphasize the need for continued and improved surveillance of IPD throughout Europe.

**Table 7:** Geographic distribution of cases and deaths of invasive pneumoccal disease for which *Streptococcus pneumoniae* serotype and disease outcome were known, Europe, 2010

Reporting country	No. (%) cases	No. (%) deaths
Austria	190 (6.5)	15 (7.9)
Belgium	1,255 (43.0)	67 (5.3)
Cyprus	3 (0.1)	0
Czech Republic	242 (8.3)	43 (17.8)
Denmark	35 (1.2)	0
Greece	20 (0.7)	1 (5.0)
Hungary	26 (0.9)	7 (26.9)
Ireland	78 (2.7)	4 (5.1)
Italy	209 (7.2)	31 (14.8)
Lithuania	3 (0.1)	0
Malta	7 (0.2)	0
Netherlands	45 (1.5)	4 (8.9)
Norway	357 (12.2)	41 (11.5)
Poland	205 (7.0)	43 (21.0)
Romania	21 (0.7)	2 (9.5)
Slovenia	224 (7.7)	6 (2.7)
Slovakia	1 (0)	0
Total	2,921 (100.0)	264 (9.04)

In conclusion, we found that older age, meningitis, non-PCV serotypes among children <5 years of age and PCV7 serotypes among persons 5–64 years of age, and penicillin nonsusceptibility were risk factors for death from IPD in Europe. The stratified analysis highlighted differences in risk for death according to *S. pneumoniae* serotype and age group. This knowledge may assist in decision making when implementing vaccination strategies as new immunization strategies are needed to tackle the considerable IPD and associated death in adults (*39*) and in designing new extended valency vaccines or protein-based pneumococcal vaccines that may confer serotype-independent immunity (*40*).
