# Correction: Vol. 21, No. 3

**DOI:** 10.3201/eid2107.C12107

**Published:** 2015-07

**Keywords:** errata, erratum, correction

The number of invasive pneumococcal disease reports was listed incorrectly in Risk Factors for Death from Invasive Pneumococcal Disease, Europe, 2010 (A. Navarro-Tornéet al.). The article has been corrected online (http://wwwnc.cdc.gov/eid/article/21/3/14-0634_article).

